# Comparative genomics of four closely related *Clostridium perfringens *bacteriophages reveals variable evolution among core genes with therapeutic potential

**DOI:** 10.1186/1471-2164-12-282

**Published:** 2011-06-01

**Authors:** Brian B Oakley, Eldin Talundzic, Cesar A Morales, Kelli L Hiett, Gregory R Siragusa, Nikolay V Volozhantsev, Bruce S Seal

**Affiliations:** 1 Poultry Microbiological Research Unit, Richard B. Russell Agricultural Research Center, Agricultural Research Service, USDA, 950 College Station Road, Athens, GA 30605, USA; 2 Department of Infectious Diseases & Center for Tropical and Emerging Global Diseases University of Georgia, Athens, GA 30306, USA; 3 State Research Center for Applied Microbiology & Biotechnology, Obolensk, Russian Federation; 4 Danisco, Inc. W227 N752 Westmound Drive Waukesha, WI 53186, USA

## Abstract

**Background:**

Because biotechnological uses of bacteriophage gene products as alternatives to conventional antibiotics will require a thorough understanding of their genomic context, we sequenced and analyzed the genomes of four closely related phages isolated from *Clostridium perfringens*, an important agricultural and human pathogen.

**Results:**

Phage whole-genome tetra-nucleotide signatures and proteomic tree topologies correlated closely with host phylogeny. Comparisons of our phage genomes to 26 others revealed three shared COGs; of particular interest within this core genome was an endolysin (PF01520, an N-acetylmuramoyl-L-alanine amidase) and a holin (PF04531). Comparative analyses of the evolutionary history and genomic context of these common phage proteins revealed two important results: 1) strongly significant host-specific sequence variation within the endolysin, and 2) a protein domain architecture apparently unique to our phage genomes in which the endolysin is located upstream of its associated holin. Endolysin sequences from our phages were one of two very distinct genotypes distinguished by variability within the putative enzymatically-active domain. The shared or core genome was comprised of genes with multiple sequence types belonging to five pfam families, and genes belonging to 12 pfam families, including the holin genes, which were nearly identical.

**Conclusions:**

Significant genomic diversity exists even among closely-related bacteriophages. Holins and endolysins represent conserved functions across divergent phage genomes and, as we demonstrate here, endolysins can have significant variability and host-specificity even among closely-related genomes. Endolysins in our phage genomes may be subject to different selective pressures than the rest of the genome. These findings may have important implications for potential biotechnological applications of phage gene products.

## Background

Concerns over the spread of antibiotic resistances among bacteria have led to a ban on antimicrobial additives to animal feeds in the European Union (EU) [1,2]. Since its enactment in 2006, the EU-wide ban on the use of antibiotics in animal feed (Regulation 1831/2003/EC) has stimulated a renewed interest in bacteriophage biology and the use of phages and/or phage gene products as alternative antibacterial agents [3,4]. Prior to the discovery and widespread use of antibiotics, bacterial infections were commonly treated by administering bacteriophages which were marketed and sold commercially for human use up until the 1940's. Bacteriophages continue to be sold in the Russian Federation and Eastern Europe as treatments for bacterial infections [5].

Recently our laboratory reported the genomic and molecular biological characteristics of two phages isolated from poultry intestinal material and poultry processing drainage water by screening for virulent *Clostridium perfringens *bacteriophages [6,7] and demonstrated efficacy of the lytic proteins encoded by the bacteriophage endolysins as a *C. perfringens *antimicrobial [8]. These phages belonged to the *Siphoviridae*, a family within the tailed phages. The tailed bacteriophages belong to the order *Caudovirales*, have icosohedral heads, contain a linear, double-stranded DNA genome that can vary from 17 to 500 kb, and represent ca. 95% of all the bacteriophages examined by electron microscope [9]. *Caudovirales *are further divided into three families based on tail morphology: phages with contractile tails are placed in the *Myoviridae*, those with short tails are members of the *Podoviridae*, and phages with a long non-contractile tail belong to the *Siphoviridae *[10,11].

*Clostridium perfringens *is a Gram-positive, spore forming, anaerobic bacterium that is the 2^nd ^leading bacterial cause of foodborne illness in the U.S., accounting for 10% of foodborne illnesses [12]. *C. perfringens *can cause food poisoning, gas gangrene (clostridial myonecrosis), enteritis necroticans, and non-foodborne gastrointestinal infections in humans and is a veterinary pathogen causing enteric diseases in both domestic and wild animals [13,14]. *C. perfringens *is considered the cause of necrotic enteritis among chickens, and although this does not generally present a threat to humans, it could potentially become a far greater problem for the poultry industry and consumers if antibiotics are withdrawn from animal feeds [13,14].

Bacteriophages have evolved a wide variety of anti-microbial compounds that can control *C. perfringens *and other pathogens and are of potential biotechnological importance. To realize this potential, it is essential to have a blueprint of the genomic machinery underlying phage-mediated bacterial lysis. Here we report the results of comparative analyses based on genome sequences of four newly isolated *C. perfringens *phages and focus on the genomic context and evolution of the phage endolysin genes.

## Results and Discussion

**Figure 1:**
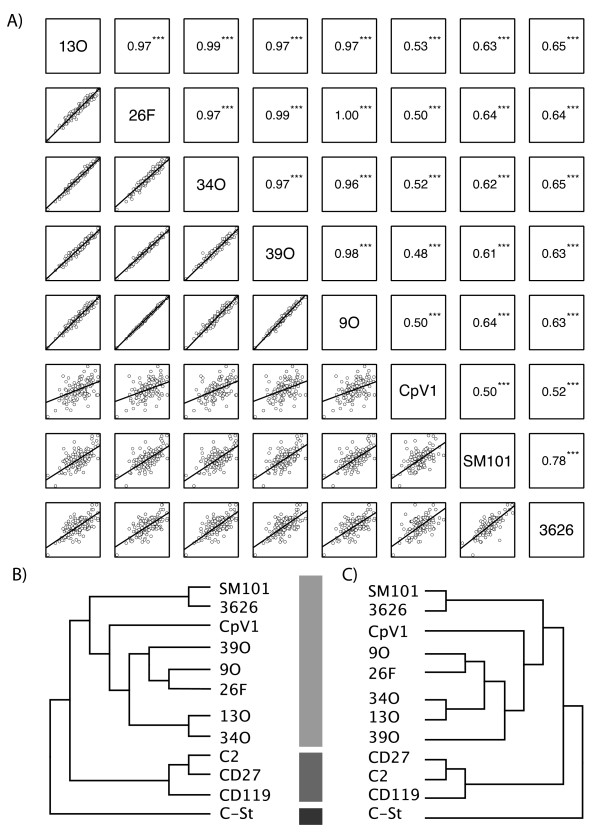
**Whole-genome comparisons of Clostridial phages**. A) Tetranucleotide-based comparisons of five genomes sequenced by our lab (ΦCP13O, ΦCP26F, ΦCP34O, ΦCP39O, ΦCP9O) to three other publicly available *C. perfringens *phage genomes (ΦCPV1, ΦSM101, Φ3626). Lower panel shows scatter plots with linear models fitted to the 256 tetra-nucleotide z-scores for each pairwise genomic comparison. Upper panel represents Pearson correlation coefficients and significance (*** = p < 0.001) of correlations. B) Cladogram representation of correlation matrix of tetranucleotide distributions from (a) with additional comparisons to *C. difficile *phages (ΦC2, ΦCD27, ΦCD119) and a *C. botulinum*-infective phage (ΦC-St). C) Proteome-based cladogram comparing the same phage genomes as in (b). Tree is based on all-versus-all sequence similarity comparisons of gene predictions using a custom analysis pipeline as fully described in the text. Note consistent and symmetrical topology of trees in (b) and (c) and consistent relationships to host as shown by shaded vertical bars.

To first determine the whole-genome relatedness of phages ΦCP9O, ΦCP13O, ΦCP26F, and ΦCP34O to each other and to other Clostridial phages, we used two approaches: correlations of tetra-nucleotide frequencies and clustering of predicted proteins based on sequence similarities. The results of both methods were consistent with each other and demonstrated close genomic relationships among our phages, more distant relationships to other Clostridial phages, and consistent correlations between phage and host phylogenies. Our phages were generally quite closely related - both techniques showed that the genomes of ΦCP34O and ΦCP13O were most closely related to each other and formed a distinct group from ΦCP26F and ΦCP9O (Figure 1). All four genomes were similar to the genome of ΦCP39O, previously published by our research group [6], and belonged to a larger clade (Figure 1B, 1C) containing ΦCPV1, a *C. perfringens *phage isolated in Russia [7]. Genomic comparisons of our phages to two other *C. perfringens *phage genomes (ΦSM101 and Φ3626), three *C. difficile*-infective phages (ΦC2, ΦCD27, and ΦCD119), and one *C. botulinum*-infective phage (ΦC-St) showed phage phylogeny closely associated with host phylogeny (Figure 1B, 1C). Our results of nearly identical topologies between tetra-nucleotide and proteomic trees is consistent with previous uses of tetra-nucleotide distributions as genomic signatures [15,16] and to infer co-evolution between virus and host [17].

### Core and accessory genomes of Clostridial phages

To determine if our phages contain a common set of genes shared with other Clostridial phages, we compared predicted ORFs based on classifications of clusters of orthologous groups (COGs) among the three host groups shown in Figure 1. COGs represent individual proteins or groups of paralogs from at least three lineages corresponding to ancient conserved domains (http://www.ncbi.nlm.nih.gov/COG/) and thus provide an informative means to compare conserved functions across genomes [18].

**Figure 2:**
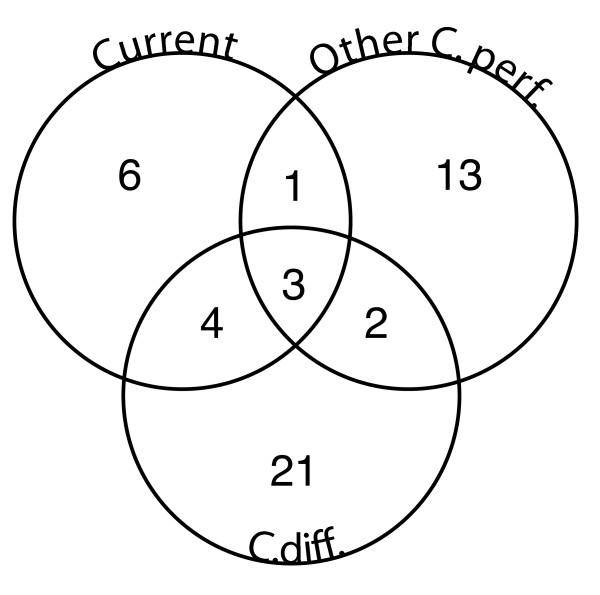
**Distribution of COGs across three host-infectivity groups**. Each circle of the Venn diagram contains all COGs belonging to the host-infectivity group; intersections represent shared COGs. Groups shown are 'Current': *C. perfringens *phage genomes published here and previously by our group (ΦCP9O, ΦCP13O, ΦCP26F, ΦCP34O, and ΦCP39O), 'Other C. perf': other *C. perfringens*-infective phages (Φ3626, ΦSM101, ΦCpV1), and 'C. diff': *C. difficile*-infective phages (ΦC2, ΦCD27, and ΦCD119). COGs were classified according to the IMG pipeline as described in the text.

Three COGs were shared among bacteriophages infecting *C. perfringens, C. difficile*, and *C. botulinum *(Figure 2). These shared COGs were COG5412, annotated as a phage-related protein of unknown function; COG0629, a single-stranded DNA-binding protein; and COG0860, a phage endolysin, N-acetylmuramoyl-L-alanine amidase (Figure 2). Endolysins, together with holins, are the key bacteriophage-encoded enzymes involved in cell wall degradation and lysis of the host and are typically transcribed from adjacent ORFs in the phage genome [8,19-21]. To better understand the evolution and natural variability of an endolysin in its genomic context, we investigated the phylogeny of the N-acetylmuramoyl-L-alanine amidase across multiple host genera and compared the phylogeny and host associations to the domain architecture of the endolysin-holin gene neighborhood.

### Statistical associations between domain architecture and phylogeny

To compare our phage sequences and domain architecture to others, we retrieved amidase sequences belonging to the pfam protein family PF01520 from 26 publicly available bacteriophage genomes (Additional file 1, Table S1) and analysed these as fully described in the methods. Bacteriophage endolysins typically contain two domains: an enzymatically active domain and a cell wall binding domain, some of which have been elucidated with crystal structures [22]. We constructed an alignment of both putative domains after building a Hidden Markov Model from representative sequences in the Conserved Domain Database belonging to PF01520 and considering only columns with >10% sequence conservation to eliminate highly variable positions and control for sequence length heterogeneity.

**Figure 3:**
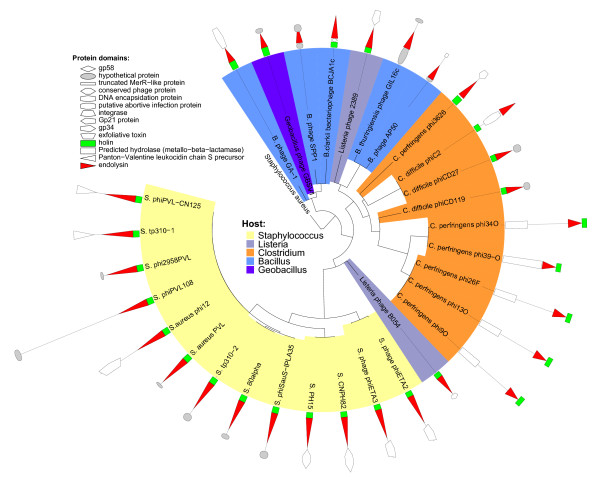
**Phylogeny of the phage-encoded peptidoglycan hydrolase, N-acetylmuramoyl-L-alanine amidase**. Sequences represent 31 phage sequences available from IMG retrieved based on annotations of COG0860 or PF01520. Sequences were aligned to a hidden Markov-model based on representative sequences from cd02696 of the CDD as described fully in the text. Domain architecture is shown by symbols on outer ringfor genes immediately upstream and downstream of the endolysin. Gene symbols are scaled according to gene lengths and coordinates as published for each genome. Clade shading scheme represents phage host as shown in the legend; significant associations between phage host and gene phylogeny were determined by UniFrac statistics based on branch length (p < 0.001) and P-tests based on topology (p < 0.001).

Several interesting conclusions could be drawn from these analyses. First, to determine whether there is a significant association between the phylogeny of the amidase protein and the identity of the bacterial host, we used the UniFrac statistic [23] which assesses unique versus shared branch lengths by host for the observed tree relative to a null distribution of host groups randomly permuted within the tree. Significant clustering by host group was found with both UniFrac (p < 0.001) and the Parsimony test (p < 0.001) which performs a similar analysis based on tree topology [24]. The association between phage lytic enzymes and host is well-known [25]; here we show a strong and statistically significant association between the N-acetylmuramoyl-L-alanine amidase phylogeny and host for a large number of phages across five host genera (Figure 3).

Second, to better understand the genomic context of the amidase protein and associated holin genes, we used the same statistical approaches to formally compare the association between the domain architecture and phylogeny of the amidase protein. The five phages sequenced by our group belong to their own clade within the amidase tree and were the only genomes in which the holin is immediately downstream of the amidase protein in the presumed direction of transcription, a reversed arrangement of the typical domain architecture (Figure 3). Interestingly, though Φ3626, ΦC2, and ΦCD27 belonged to a sister clade, this domain architecture was unique even among these other Clostridial phages (Figure 3). To confirm this domain architecture for our phages, we re-sequenced the appropriate regions of Φ9O, Φ13O, Φ26F, Φ34O, and several other phage isolates, all of which shared the amidase-holin arrangement. Holin genes were identified using multiple sequence-similarity approaches as described in detail in the methods, and included identifications of transmembrane domains. The association between gene phylogeny and domain architecture was strongly significant as determined by UniFrac (p < 0.001) and P tests (p < 0.001).

Because lysis of bacterial cells generally requires both an endolysin and a holin - membrane disruption (the function of the holin) is considered to be requisite for the endolysin to attack the peptidoglycan [19] - understanding the phylogenetics and genomic context of these genes are important milestones to develop biotechnological applications. The unusual domain architecture we observed suggests that either the typical gene order or the reverse is a successful evolutionary strategy. The transcriptional regulation of these genes in our phages remains unknown, but searches for transcriptional promoters and terminators using BPROM (Softberry, Inc., Mount Kisco, NY, USA; http://linux1.softberry.com/berry.phtml) and TransTerm (http://nbc3.biologie.uni-kl.de) did not find either within the regions of our endolysin and holin genes; these genes may be co-transcribed. Efficacy of the endolysin as recently demonstrated for phages ΦCP26F and ΦCP39O [8] could potentially be improved by successful holin purification.

### Genomic arrangement and context of orthologs

**Figure 4:**
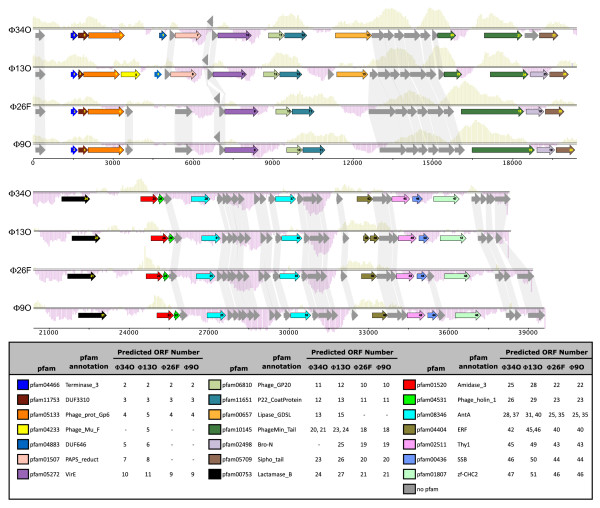
**Genome maps for phage ΦCP9O, ΦCP13O, ΦCP26F, and ΦCP34O**. Arrows are shown in presumed direction of transcription. Grey arrows indicate genes of unknown homology or function, colored arrows indicate genes belonging to pfam families or clusters of orthologous groups (COGs) as classified by IMG. Functional categorizations of pfam designations are shown in the legend. For predicted ORFs with unknown function, sequence similarity clustering at a 40% similarity cutoff is indicated by shading.

Twenty-one pfam families were identified among the four phage genomes (Figure 4). Of these, only one, PF04233, annotated as a homolog of phage Mu protein gp30, was found in only one genome (ΦCP13O). Three other pfams were found in 2-3 genomes and were absent from the other(s). A prophage antirepressor (PF02498) was present and 100% identical in the genomes of ΦCP9O, ΦCP13O, and ΦCP26F, but, interestingly, a syntenous protein of ΦCP34O (gene product 22, Figure 4), had no significant sequence similarity to these sequences based on pairwise blastp and no significant matches to any pfam domains. Similarly, 3'-phosphoadenosine 5'-phosphosulfate sulfotransferase (PAPS reductase)/FAD synthetase (pfam01507) genes were present in the genomes of ΦCP13O and ΦCP34O with 100% pairwise sequence similarity, but approximately syntenous ORFs in the genomes of ΦCP9O and ΦCP26F had no significant blastp similarity to COG0175 and did not match any pfam domains. The majority of pfams (17/21) were present in all four bacteriophage genomes (Figure 4). Detailed statistics for each genome are shown in Additional file 2, Table S3.

### Conservation and variability of core genome

**Figure 5:**
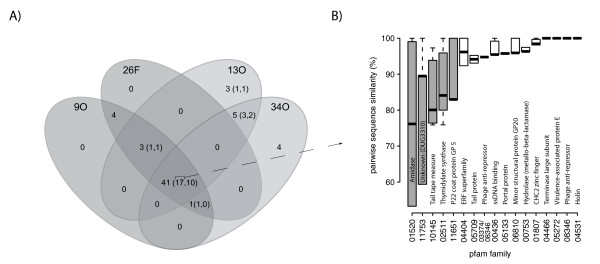
**Core and accessory genomes of ΦCP9O, ΦCP13O, ΦCP26F, and ΦCP34O**. A) Shared and unique genes based on sequence similarity clustering of genes belonging to pfam families or clusters of orthologous groups (COGs) as classified by IMG and shown in Figure 4. Clustering was performed using CD-HIT with a 40% sequence similarity cutoff as described in the text. Each circle of the Venn diagram contains all genes belonging to the host-infectivity group; intersections represent shared genes. Numbers of outside of parentheses indicate total number of ORFs, numbers inside parentheses indicate numbers of genes belonging to known pfam families and COGs respectively. B) Distributions of pairwise sequence similarity for the 17 core pfam families shown in (a). Within each pfam, all possible pairwise similarities were calculated for each gene from the four phages compared in (a) and ΦCP39O. Genes belonging to the five pfam families shown in grey were defined as a variable core genome and boxes in white defined as a conserved core genome.

To investigate shared genes in more detail and to classify the majority of predicted ORFs which were not assigned to COGs or pfams, we next compared the distributions of pfams and sequence-similarity groups derived by clustering of all predicted ORFs across all four genomes to determine a core and accessory genome (Figure 5). Most gene clusters (41/61) were shared by all four genomes on the basis of sequence similarity (Figure 5a). Of the 17 pfam families that were common to all four genomes, we considered 12 to represent a 'conserved core genome', and five to represent a 'variable core genome' based on pairwise sequence similarities (Figure 5b). The five pfam families in the core genome containing highly variable genes were: PF01520, the N-acetylmuramoyl-L-alanine amidase (COG0860); PF11753 of unknown function; PF10145, a tail tape measure protein (COG5412); PF 02511, a thymidylate synthase (COG1351) involved in nucleotide transport and metabolism; and PF11651, a P22 coat protein (Figure 5b).

In the conserved core genome, genes within each of the 12 pfam families were very similar to each other, with a maximum pairwise sequence difference of 8% based on amino acid alignments with bl2seq (Figure 5b). Genes belonging to these 12 pfam families were involved in the following functions: tail protein, phage anti-repressor, ssDNA binding, portal protein, minor structural protein GP20, hydrolase, CHC2 zinc finger, terminase large subunit, virulence-associated protein E, and the holin (Figure 5b).

The holin genes were among the most conserved, with 100% identity among all sequences, and the amidase genes were the most variable (Figure 5b), suggesting these two genes are subject to very different rates of evolution despite their colocation in the genome and paired function in the lytic cycle. Holins target the relatively invariable cytoplasmic membrane, while phage endolysins recognize and degrade the cell wall, which is highly variable. It has been suggested that holins may function as a type of lysis clock, governing the timing of lysis of the host [26]. As the primary determinant of the length of the infective cycle, holins can be considered to experience stabilizing selection as there are opposing fitness advantages to extending the vegetative cycle and allowing phage replication versus lysing the host to release progeny phage to infect new host cells [19]. In contrast, the phage endolysins generally contain an enzymatically active domain and a cell-wall binding domain which recognizes highly-specific ligands on the host cell surface [27], and thus each domain is under strong directional selective pressures. Our data clearly show strong sequence conservation of the holin protein, and very distinct sequence types within the associated amidase for a group of closely related phages.

### Detailed sequence comparisons of variable core genome and association with host genotype

For the four pfam families with known functions in the variable core genome, multiple-sequence alignments of the four genomes presented here and ΦCP39O sequenced previously by our group [6] revealed some striking differences in amino acid length and content. For all four proteins, two very distinct sequence types were represented.

**Figure 6:**
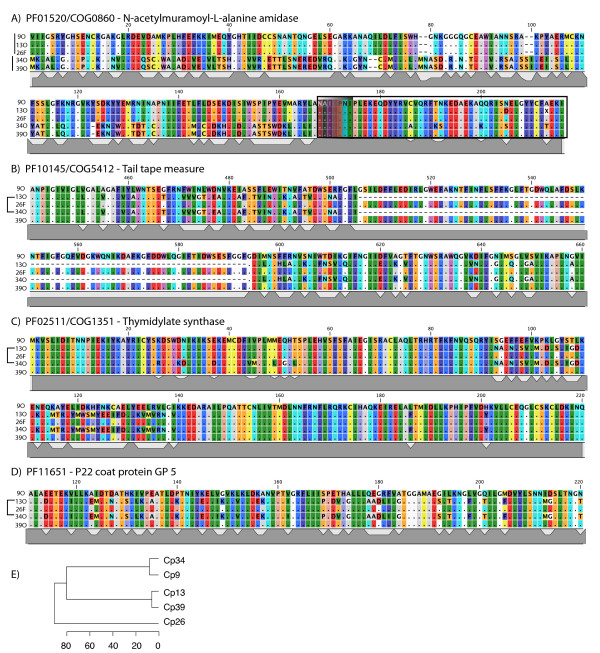
**Multiple-sequence alignments for each of the four pfam families of the variable core genome with known functions**. Dots represent conserved positions relative to the first sequence, topologies of sequence-similarity groupings are shown to the left of each alignment. Alignments illustrate distinct sequence types defined by sequence variation and major deletions/insertions within each gene for A) PF01520/COG0860, N-acetylmuramoyl-L-alanine amidase, B) PF10145/COG5412, tape measure protein, C) PF02511/COG1351, thymidylate synthase, and D) PF11651, P22 coat protein. Whole genome relationships of host *C. perfringens *as determined by rep-PCR are shown in (E). Scale represents % dissimiliarity. For each gene, topologies of neighbor-joining trees are outlined to the left of the alignment. For A and C, alignments of entire protein are shown; for B, the entire protein was 780 AA, not shown is 220 AA N-terminal deletion for ΦCP13O and ΦCP34O which is encoded by another ORF immediately upstream. Alignments were done using MUSCLE with default parameters as described in the text. Inferred EAD (N-terminal) and CBD (C-terminal) domains of the amidase joined by a linker region are designated by boxes in (A).

For the amidase, ΦCP39O and ΦCP34O were most closely related and clearly distinct from the sequence types of ΦCP9O, ΦCP13O, and ΦCP26F (Figure 6a). The N-terminal portion of the protein from amino acid residues 1-166 of the multiple sequence alignment was the most variable portion of the protein (Figure 6a), and corresponds within approximately 5 residues to the enzymatically active domain (EAD) determined structurally and experimentally [22] for the endolysin from *Listeria *phage 2389 (NC_003291). The C-terminal portion of the protein, corresponding to the cell wall binding domain (CBD) of *Listeria *phage 2389 is more conserved than the EAD in our phages (Figure 6a).

The tape measure proteins (PF10145/COG5412) of ΦCP26F, ΦCP9O, and ΦCP39O were all 780AA long and 96% similar to each other and quite different from those of ΦCP13O and ΦCP34O. The tape measure proteins of ΦCP34O and ΦCP13O were 95% similar to each other, but only 473AA residues in length with a 225 AA N-terminal portion of the protein encoded by another ORF immediately upstream in the genome. For the portion of the protein encoded by a single reading frame, alignments of these five sequences revealed a deletion of 89 residues in the tape measure proteins of ΦCP34O and ΦCP13O (Figure 6b). Whether these represent gene fissions or fusions, or insertions or deletions relative to the ancestral state remains unknown, as do the consequences for the structure and function of the protein, but clearly these questions warrant further study.

For the thymidylate synthase (PF02511/COG1351), the phage relatedness patterns were the same as for the tape measure protein, with ΦCP34O and ΦCP13O containing a similar genotype distinct from that of ΦCP9O and ΦCP39O, largely defined by a variable region from residues 93-139 (Figure 6c). Similarly, the P22 coat proteins (PF11651) of ΦCP13O and ΦCP34O were distinct from those shared by ΦCP9O, ΦCP26F, and ΦCP39O (Figure 6d).

In contrast to these groupings, genomic fingerprints of the *C. perfringens *host based on rep-PCR defined three main host groups: 1) Cp34O and Cp9O, 2) Cp13O and Cp39O, and 3) Cp26F as a more distantly related group (Figure 6e). Interestingly, the single gene phage similarities based on the tape measure protein, the thymidylate synthase, and the coat protein reflected the whole-genome groupings shown in Figure 1 with ΦCP13O and ΦCP34O most similar to each other and ΦCP9O, ΦCP26F, and ΦCP39O forming a separate group. In contrast, sequence similarities based on the amidase protein were not concordant with the other genes in the core genome or the whole-genome clustering. Based on these data, we concluded that the selective pressures on the amidase genes for these phages are somehow unique from the rest of the genome. This result may have important implications for potential biotechnological applications in which amidase proteins are used separately or together with other gene products such as holins for bacterial control.

### Endolysin protein structure

**Figure 7:**
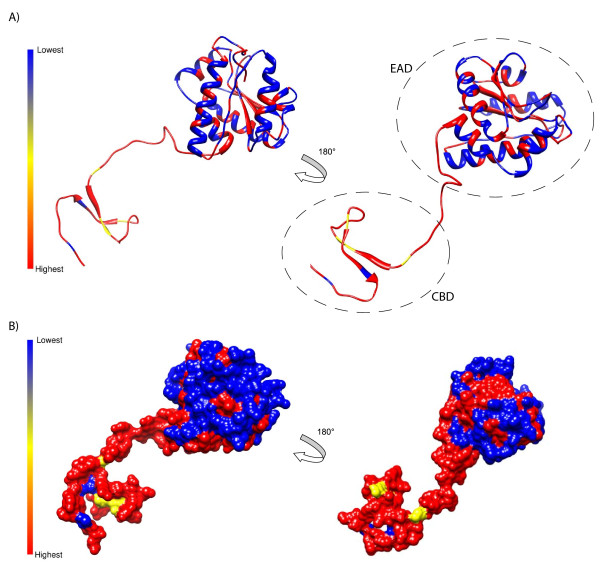
**Three-dimensional structures indicating amino acid conservation for Clostridium bacteriophages ΦCP9O, ΦCP13O, ΦCP26F, ΦCP34O, and ΦCP39O**. Percent sequence conservation among the five phages is shown from highest 100% (red) to lowest 60% (blue) for cartoon (A) and surface based (B) models. Dashed circles illustrate the putative enzymatically-active domain (EAD) and the putative cell-wall binding domain (CBD). Low sequence conservation represents regions under high diversifying selection, primarily in the enzymatically-active domain.

To investigate the association between the sequence variability of our phages and the structure of the EAD and the CBD of the amidase, we constructed a structural model using as a template a related structure from a *Listeria *phage (PDB; 1XOV) previously solved with crystallography [22]. Comparative modelling of bacteriophage lytic enzymes is becoming a common tool to inform the development of phage lysin-based biocontrol agents [28]. N-acetylmuramoyl-L-alanine amidases are one of at least six types of phage endolysins and attack the amide bonds between the amino sugar MurNAc and L-Ala of the cross-linking peptide stem in the peptidoglycan layer of the host cell wall [21]. The specificity of the enzyme is thought to be due to recognition of specific ligands on the host cell surface by the CBD [21]. Our modeling revealed that the enzymatic core is formed by a twisted, six-stranded β-sheet flanked by six helices (α1-α6) linked through a loop region to the cell wall binding domain which consists of two anti-parallel β-sheets (Figure 7). The areas of highest sequence conservation were concentrated in the CBD and the central portion of the enzymatic domain (Figure 7). Several point mutations within the CBD may contribute to its specificity, but interestingly, for our phages, the N-terminal EAD was much more variable than the CBD, suggesting much higher diversifying selective pressures on this portion of the protein.

## Conclusions

Comparisons of genome sequences from four newly isolated *C. perfringens *phages and related sequences previously published has provided new insights into genomic conservation and variability. Sequence and structural variability of the endolysin EAD may have important implications for the potential to target specific strains of pathogenic bacteria. Sequence and structural conservation of the CBD suggests the potential to tailor specificity for detection and differentiation of target cell populations, extending previous work [29]. Holins and endolysins represent conserved functions across divergent phage genomes and, as we demonstrate here, endolysins can have significant variability and host-specificity even among closely-related genomes. Endolysins in our phage genomes may be subject to different selective pressures than the rest of the genome, with important implications for potential biotechnological applications of these phages and their gene products.

## Methods

### Bacteriophage Genome Sequencing

Purification and propagation of bacteriophages and subsequent genomic DNA purification was carried out as previously described in detail [6]. Sequencing of the bacteriophage genomes was completed by MWG Biotech, Inc High Point, NC by Sanger and pyrosequencing to 14-fold redundancy that included primer-walking to fill gaps.

### Genome Annotations and comparisons

Gene predictions and genome annotations were performed with the IMG pipeline [30], which uses a combination of Hidden Markov Models and sequence similarity searches. Briefly, gene predictions were performed with GeneMark [31] and then compared to COG PSSMs obtained from the CDD database [32], searched against the KEGG genes database [33] with BLASTp, and then searched against the Pfam [34] and TIGRfam [35] databases using BLAST prefiltering and subsequent comparison to HMMs using hmmsearch [36]. To compare the phylogeny and protein domain architecture of phage-encoded endolysin and holin genes, genomes of 26 bacteriophage were retrieved from IMG (Additional file 1, Table S1) based on top ortholog hits to COG0860. Genome accession numbers and basic summary statistics are shown in Additional file 1, Table S2. Gene predictions, annotations, and genome coordinates are listed for each genome in Additional file 2, Table S3.

Tetra-nucleotide distributions for Clostridial phage genomes and correlation coefficients between genomes were calculated with TETRA [37]. Correlation coefficients were transformed to a dissimilarity matrix for tree construction using the hierarchical clustering algorithm hclust in R [38], which was also used to generate dendrograms and visualize tetra-nucleotide distributions. Proteomic comparisons of Clostridial phage genomes was performed with a custom analysis pipeline we constructed using CD-HIT [39] for clustering of predicted ORFs. Output was parsed with a series of perl scripts, and dendrograms constructed in mothur [40] using the Jaccard similarity index. COG and pfam designations from IMG for each genome were used to determine shared and accessory functions across the 12 Clostridial phage genomes. To construct genome maps, annotated genome files were transferred to Artemis [41] and genome maps constructed with DNA Plotter [42]. rep-PCR of host genomes was performed as previously described [43].

### Tree construction

Bacteriophage endolysin sequences belonging to COG0860 and/or PF01520 were retrieved from IMG and Genbank genomes using BioPerl. A seed alignment of 100 representative sequences belonging to conserved domain cd0269 in the CDD (10) was used to build a Hidden-Markov profile and the phage sequences shown in Figure 3 were aligned to this HMM model using Hmmer 3.0 (14). Aligned sequences were imported into ARB [44] where trees were constructed with neighbor-joining and maximum-likelihood methods restricted to columns sharing at least 10% sequence identity. When identical topologies were obtained with both methods, tree files were exported and visualized with ITOL [45]. The significance of associations between phylogeny and host, and phylogeny and protein domain architecture was assessed with UniFrac [23] and Parsimony tests [24], which use a Monte Carlo approach to compare observed phylogenies with a null model derived from random permutations.

### Designation and comparisons of core versus accessory genomes

Shared and unique genes, COGs, and pfams were determined by two methods. First, the same analysis pipeline described above was used to group predicted ORFs on the basis of sequence similarity as determined by CD-HIT [39]. Second, classifications from IMG were used to determine shared and unique COGs and pfam families. The similarity of genes belonging to each pfam family in the core genome was determined by pairwise blastp implemented with the bl2seq algorithm in a perl script.

### Structural Modeling

The 3D structure of the endolysin from ΦCP26F (ORF22, pfam01520) was modeled using the HHpred server with default settings [46]. Briefly, the HHpred method is specialized in remote homology detection using hidden Markow models (HMMs) built from PSI-BLAST profiles and secondary structures. The crystal structure of *Listeria *PlyPSA (Protein Data Bank code 1XOV chain A, [22]) was used as a template since it had the highest sequence and secondary structure scores. Lastly, a 3D model was generated using MODELLER [47] and visualized using the UCSF Chimera molecular analysis program [48]. Sequence conservation among our five phages was calculated using the mavPercentConservation method based on the AL2CO algorithm [48] which performs calculations in two steps. First, amino acid frequencies at each position are estimated and then the conservation index is calculated from these frequencies. The results were then mapped to the predicted protein structure of ΦCP26F using the following color parameters: lowest (60%) and highest (100%) sequence conservation.

## Authors' contributions

BBO annotated genomes, analyzed data, and wrote the ms; ET performed the structural modeling and contributed to the ms; CAM provided technical support; NVV and KLH contributed to genome sequencing efforts; GRS initiated the study, isolated the phages, and performed rep-PCR; and BSS oversaw the genome sequencing and supervised the project. All authors read and approved the final manuscript.

## Supplementary Material

Additional file 1 **Additional_File1_TableS1-S2.pdf**. Genome accession numbers and summary statistics. Click here for file

Additional file 2 **Additional_File2_TableS3.xls**. Gene predictions, annotations, and genome coordinates. Click here for file

## References

[B1] Bedford M Removal of antibiotic growth promoters from poultry diets: implications and strategies to minimize subsequent problems World Poultry Science Journal 2000 56 347 365 10.1079/WPS20000024

[B2] Castanon JI History of the Use of Antibiotic as Growth Promoters in European Poultry Feeds Poultry Science 2007 86 2466 2471 10.3382/ps.2007-00249 17954599

[B3] Merril CR Biswas B Carlton R Jensen NC Creed GJ Zullo S Adhya S Long-circulating bacteriophage as antibacterial agents Proc Natl Acad Sci USA 1996 93 3188 3192 10.1073/pnas.93.8.3188 8622911 PMC39580

[B4] Liu J Dehbi M Moeck G Arhin F Bauda P Bergeron D Callejo M Ferretti V Ha N Kwan T Antimicrobial drug discovery through bacteriophage genomics Nat Biotechnol 2004 22 185 191 10.1038/nbt932 14716317

[B5] Sulakvelidze A Alavidze Z Morris J Antimicrobial Agents and Chemotherapy Bacteriophage therapy 2001 45 649 659 10.1128/AAC.45.3.649-659.2001 PMC90351 11181338

[B6] Seal BS Fouts DE Simmons M Garrish JK Kuntz RL Woolsey R Schegg KM Kropinski AM Ackermann HW Siragusa GR Clostridium perfringens bacteriophages PhiCP39O and PhiCP26F: genomic organization and proteomic analysis of the virions Arch Virol 2010 21 21 10.1007/s00705-010-0812-z PMC4127328 20963614

[B7] Volozhantsev NV Verevkin VV Bannov VA Krasilnikova VM Myakinina VP Zhilenkov EL Svetoch EA Stern NJ Oakley BB Seal BS The genome sequence and proteome of bacteriophage PhiCPV1 virulent for Clostridium perfringens Virus Res 2010 10.1016/j.virusres.2010.11.012 21144870

[B8] Simmons M Donovan DM Siragusa GR Seal BS Recombinant expression of two bacteriophage proteins that lyse clostridium perfringens and share identical sequences in the C-terminal cell wall binding domain of the molecules but are dissimilar in their N-terminal active domains J Agric Food Chem 2010 58 10330 10337 10.1021/jf101387v 20825156 PMC4115659

[B9] Ackermann H 5500 Phages examined in the electron microscope Archives of Virology 2007 152 227 243 10.1007/s00705-006-0849-1 17051420

[B10] Ackermann H Bacteriophage observations and evolution Research in Microbiology 2003 154 245 251 10.1016/S0923-2508(03)00067-6 12798228

[B11] Ackermann H Calender R Classification of Bacteriophages The Bacteriophages 2006 Oxford: Oxford University Press 8 16

[B12] Scallan E Hoekstra RM Angulo FJ Tauxe RV Widdowson MA Roy SL Jones JL Griffin PM Foodborne illness acquired in the United States-major pathogens Emerg Infect Dis 2011 17 7 15 21192848 10.3201/eid1701.P11101 PMC3375761

[B13] Sawires YS Songer JG Clostridium perfringens: insight into virulence evolution and population structure Anaerobe 2006 12 23 43 10.1016/j.anaerobe.2005.10.002 16701609

[B14] Van Immerseel F De Buck J Pasmans F Huyghebaert G Haesebrouck F Ducatelle R Clostridium perfringens in poultry: an emerging threat for animal and public health Avian Pathol 2004 33 537 549 10.1080/03079450400013162 15763720

[B15] Pride DT Meinersmann RJ Wassenaar TM Blaser MJ Evolutionary implications of microbial genome tetranucleotide frequency biases Genome Res 2003 13 145 158 10.1101/gr.335003 12566393 PMC420360

[B16] Teeling H Meyerdierks A Bauer M Amann R Glockner FO Application of tetranucleotide frequencies for the assignment of genomic fragments Environ Microbiol 2004 6 938 947 10.1111/j.1462-2920.2004.00624.x 15305919

[B17] Pride DT Wassenaar TM Ghose C Blaser MJ Evidence of host-virus co-evolution in tetranucleotide usage patterns of bacteriophages and eukaryotic viruses BMC Genomics 2006 7 8 10.1186/1471-2164-7-8 16417644 PMC1360066

[B18] Tatusov RL Koonin EV Lipman DJ A genomic perspective on protein families Science 1997 278 631 637 10.1126/science.278.5338.631 9381173

[B19] Wang IN Smith DL Young R Holins: the protein clocks of bacteriophage infections Annu Rev Microbiol 2000 54 799 825 10.1146/annurev.micro.54.1.799 11018145

[B20] Rigden DJ Jedrzejas MJ Galperin MY Amidase domains from bacterial and phage autolysins define a family of gamma-D,L-glutamate-specific amidohydrolases Trends Biochem Sci 2003 28 230 234 10.1016/S0968-0004(03)00062-8 12765833

[B21] Loessner MJ Bacteriophage endolysins--current state of research and applications Curr Opin Microbiol 2005 8 480 487 10.1016/j.mib.2005.06.002 15979390

[B22] Korndorfer IP Danzer J Schmelcher M Zimmer M Skerra A Loessner MJ The crystal structure of the bacteriophage PSA endolysin reveals a unique fold responsible for specific recognition of Listeria cell walls J Mol Biol 2006 364 678 689 10.1016/j.jmb.2006.08.069 17010991

[B23] Lozupone C Knight R UniFrac: a new phylogenetic method for comparing microbial communities Appl Environ Microbiol 2005 71 8228 8235 10.1128/AEM.71.12.8228-8235.2005 16332807 PMC1317376

[B24] Martin AP Phylogenetic approaches for describing and comparing the diversity of microbial communities Appl Environ Microbiol 2002 68 3673 3682 10.1128/AEM.68.8.3673-3682.2002 12147459 PMC124012

[B25] Bernhardt TG Wang IN Struck DK Young R Breaking free: "protein antibiotics" and phage lysis Res Microbiol 2002 153 493 501 10.1016/S0923-2508(02)01330-X 12437210

[B26] Grundling A Manson MD Young R Holins kill without warning Proc Natl Acad Sci USA 2001 98 9348 9352 10.1073/pnas.151247598 11459934 PMC55423

[B27] Loessner MJ Kramer K Ebel F Scherer S C-terminal domains of Listeria monocytogenes bacteriophage murein hydrolases determine specific recognition and high-affinity binding to bacterial cell wall carbohydrates Mol Microbiol 2002 44 335 349 10.1046/j.1365-2958.2002.02889.x 11972774

[B28] Henry M Coffey A O'Mahony JM Sleator RD Comparative modelling of LysB from the mycobacterial bacteriophage Ardmore Bioengineered Bugs 2011 2 1 8 10.4161/bbug.2.1.14315 21636995

[B29] Schmelcher M Shabarova T Eugster MR Eichenseher F Tchang VS Banz M Loessner MJ Rapid multiplex detection and differentiation of Listeria cells by use of fluorescent phage endolysin cell wall binding domains Appl Environ Microbiol 2010 76 5745 5756 10.1128/AEM.00801-10 20622130 PMC2935047

[B30] Markowitz VM Mavromatis K Ivanova NN Chen IM Chu K Kyrpides NC IMG ER: a system for microbial genome annotation expert review and curation Bioinformatics 2009 25 2271 2278 10.1093/bioinformatics/btp393 19561336

[B31] Besemer J Lomsadze A Borodovsky M GeneMarkS: a self-training method for prediction of gene starts in microbial genomes. Implications for finding sequence motifs in regulatory regions Nucleic Acids Res 2001 29 2607 2618 10.1093/nar/29.12.2607 11410670 PMC55746

[B32] Marchler-Bauer A Lu S Anderson JB Chitsaz F Derbyshire MK Deweese-Scott C Fong JH Geer LY Geer RC Gonzales NR CDD: a Conserved Domain Database for the functional annotation of proteins Nucleic Acids Res 2010 24 24 10.1093/nar/gkq1189 PMC3013737 21109532

[B33] Ogata H Goto S Sato K Fujibuchi W Bono H Kanehisa M KEGG: Kyoto Encyclopedia of Genes and Genomes Nucleic Acids Res 1999 27 29 34 10.1093/nar/27.1.29 9847135 PMC148090

[B34] Bateman A Birney E Cerruti L Durbin R Etwiller L Eddy SR Griffiths-Jones S Howe KL Marshall M Sonnhammer EL The Pfam protein families database Nucleic Acids Res 2002 30 276 280 10.1093/nar/30.1.276 11752314 PMC99071

[B35] Haft DH Loftus BJ Richardson DL Yang F Eisen JA Paulsen IT White O TIGRFAMs: a protein family resource for the functional identification of proteins Nucleic Acids Res 2001 29 41 43 10.1093/nar/29.1.41 11125044 PMC29844

[B36] Eddy SR Profile hidden Markov models Bioinformatics 1998 14 755 763 10.1093/bioinformatics/14.9.755 9918945

[B37] Teeling H Waldmann J Lombardot T Bauer M Glockner F TETRA: a web-service and a stand-alone program for the analysis and comparison of tetranucleotide usage patterns in DNA sequences BMC Bioinformatics 2004 5 163 10.1186/1471-2105-5-163 15507136 PMC529438

[B38] Team RDC R: A language and environment for statistical computing Book R: A language and environment for statistical computing (Editor ed.^eds.) 2008 City: R Foundation for Statistical Computing

[B39] Li W Godzik A Cd-hit: a fast program for clustering and comparing large sets of protein or nucleotide sequences Bioinformatics 2006 22 1658 1659 10.1093/bioinformatics/btl158 16731699

[B40] Schloss PD Westcott SL Ryabin T Hall JR Hartmann M Hollister EB Lesniewski RA Oakley BB Parks DH Robinson CJ Introducing mothur: open-source, platform-independent, community-supported software for describing and comparing microbial communities Appl Environ Microbiol 2009 75 7537 7541 10.1128/AEM.01541-09 19801464 PMC2786419

[B41] Rutherford K Parkhill J Crook J Horsnell T Rice P Rajandream MA Barrell B Artemis: sequence visualization and annotation Bioinformatics 2000 16 944 945 10.1093/bioinformatics/16.10.944 11120685

[B42] Carver T Thomson N Bleasby A Berriman M Parkhill J DNAPlotter: circular and linear interactive genome visualization Bioinformatics 2009 25 119 120 10.1093/bioinformatics/btn578 18990721 PMC2612626

[B43] Siragusa GR Danyluk MD Hiett KL Wise MG Craven SE Molecular subtyping of poultry-associated type A Clostridium perfringens isolates by repetitive-element PCR J Clin Microbiol 2006 44 1065 1073 10.1128/JCM.44.3.1065-1073.2006 16517895 PMC1393103

[B44] Ludwig W Strunk O Westram R Richter L Meier H Yadhukumar Buchner A Lai T Steppi S Jobb G ARB: a software environment for sequence data Nucleic Acids Res 2004 32 1363 1371 10.1093/nar/gkh293 14985472 PMC390282

[B45] Letunic I Bork P Interactive Tree Of Life (iTOL): an online tool for phylogenetic tree display and annotation Bioinformatics 2007 23 127 128 10.1093/bioinformatics/btl529 17050570

[B46] Soding J Biegert A Lupas AN The HHpred interactive server for protein homology detection and structure prediction Nucleic Acids Res 2005 33 W244 248 10.1093/nar/gki408 15980461 PMC1160169

[B47] Eswar N Webb B Marti-Renom MA Madhusudhan MS Eramian D Shen MY Pieper U Sali A Comparative protein structure modeling using Modeller Curr Protoc Bioinformatics 2006 Chapter 5 Unit 5 6 18428767 10.1002/0471250953.bi0506s15 PMC4186674

[B48] Pettersen EF Goddard TD Huang CC Couch GS Greenblatt DM Meng EC Ferrin TE UCSF Chimera--a visualization system for exploratory research and analysis J Comput Chem 2004 25 1605 1612 10.1002/jcc.20084 15264254

