# Supplementary material for: Comparative genomics of four closely related Clostridium perfringens bacteriophages reveals variable evolution among core genes with therapeutic potential
Source: BMC Genomics. 2011 Jun 1;12:282. doi: 10.1186/1471-2164-12-282 (PMC3118219; doi:10.1186/1471-2164-12-282)
Supplement: Additional file 1 — Additional_File1_TableS1-S2.pdf. Genome accession numbers and summary statistics. [file 1471-2164-12-282-S1.PDF]

Table S1. Identities and accession numbers of phages used for comparative analyses.

IMG ID	full name	GB_acc
**641210038**	Staphylococcus phage tp310-2 provirus	NC_009762
**641209970**	Staphylococcus phage tp310-1 provirus	NC_009761
**642978062**	Staphylococcus phage phiSauS-IPLA35	NC_011612
**641204912**	Staphylococcus phage phiPVL108	NC_008689
**645046073**	Staphylococcus phage phiPVL-CN125	NC_012784
**641206518**	Staphylococcus phage phiETA3	NC_008799
**641206450**	Staphylococcus phage phiETA2	NC_008798
**642976413**	Staphylococcus phage phi2958PVL	NC_011344
**641205308**	Staphylococcus phage PH15	NC_008723
**641205245**	Staphylococcus phage CNPH82	NC_008722
**641208734**	Staphylococcus phage 80alpha	NC_009526
**638297097**	Staphylococcus aureus phage phi 12	NC_004616
**638284543**	Staphylococcus aureus bacteriophage PVL	NC_002321
**641510114**	Listeria phage B054	NC_009813
**638290276**	Listeria phage 2389	NC_003291
**641202568**	Geobacillus phage GBSV1	NC_008376
**642976997**	Clostridium phage phiCD27	NC_011398
**641207493**	Clostridium phage phiC2	NC_009231
**642976321**	Clostridium phage 39-O	NC_011318
**638319424**	Clostridium difficile bacteriophage phi CD119	NC_007917
**638292480**	Bacteriophage phi3626	NC_003524
**638312133**	Bacillus thuringiensis phage GIL16c	NC_006945
**638295403**	Bacillus phage SPP1	NC_004166
**638286350**	Bacillus phage GA-1	NC_002649
**642977436**	Bacillus phage AP50	NC_011523
**638310406**	Bacillus clarkii bacteriophage BCJA1c	NC_006557

Table S2. Basic genome characteristics and accession numbers.

Genome	Accession number	Genes	w/ Func Pred	KEGG Orthology (KO)	COG	Pfam	GC Perc	Bases
ΦCP13O	JF67208	55	23	2	14	24	30.0%	38326
ΦCP26F	GQ443085	49	18	2	11	18	30.0%	39188
ΦCP34O	JF767209	52	20	2	12	21	31.0%	38309
ΦCP9O	JF767210	49	18	2	11	18	30.0%	39594
